# Intergenic Interactions of ESR1, GSTO1 and AGER and Risk of Dementia in Community-Dwelling Elderly (SADEM Study)

**DOI:** 10.3390/genes15111395

**Published:** 2024-10-29

**Authors:** Teresa Juárez-Cedillo, Nancy Martínez-Rodríguez, Enrique Juárez-Cedillo, Alfredo Ramirez, Alan Suerna-Hernández

**Affiliations:** 1 Unidad de Investigación Médica en Epidemiológica Clínica, Centro Médico Nacional Siglo XXI, Instituto Mexicano del Seguro Social, Mexico City 06720, CP, Mexico; 2 Epidemiology, Endocrinology and Nutrition Research Unit, Hospital Infantil de México Federico Gómez, Ministry of Health (SSA), Mexico City 06720, CP, Mexico; amr70@hotmail.com; 3 Facultad de Medicina, Universidad Nacional Autónoma de Mexico, Mexico City 04360, CP, Mexico; ejcedillo@yahoo.com.mx (E.J.-C.); alansuerna@yahoo.com.mx (A.S.-H.); 4 Division of Neurogenetics and Molecular Psychiatry, Department of Psychiatry and Psychotherapy, University of Cologne, 50923 Köln, Germany; alfredo.ramirez-ziniga@uk-koeln.de

**Keywords:** dementia, MDR, polymorphism, gen-gen interaction, *GSTO1*, *ESR1*, *AGER*, bioinformatic

## Abstract

Background: Dementia causes the loss of functional independence, resulting in a decrease in the quality of life of those who suffer from it. Aims: This study aimed to investigate the interactions influencing susceptibility to the development of dementia through multifactor dimensionality reduction (MDR). Methods: the study population was made up of 221 cases and 534 controls. We performed an MDR analysis as well as a bioinformatic analysis to identify interactions between the genes *GSTO1*_rs4925, *AGER*_rs2070600, and *ESR1*_rs3844508 associated with susceptibility to dementia. Results: We observed associations between the polymorphism of GSTO1 and risk of dementia for the site rs4925 with the recessive model (OR = 1.720, 95% CI = 1.166–2.537 *p* = 0.006). Similarly, the site AGER rs2070600 showed risk of dementia with an additive model of inheritance (OR = 7.278, 95% CI = 3.140–16.868; *p* < 0.001). Furthermore, we identified the best risk model with a high precision of 79.6% that, when combined with three environmental risk factors, did not give an OR = 26.662 95%CI (16.164–43.979) with *p* < 0.001. Conclusions: The MDR and bioinformatics results provide new information on the molecular pathogenesis of dementia, allowing identification of possible diagnostic markers and new therapeutic targets.

## 1. Introduction

Dementia involves the progressive loss of cognitive function over the years and is one of the most common disorders of aging, leading to a decrease in the quality of life of patients that limits daily activity. It is a multifactorial disease, and both clinical and genetic factors seem to be involved in its pathogenesis. Among the factors involved are the action of β-amyloid and oligomers of the following peptides: [1] oxidative stress [2] and proinflammatory cytokines produced by the activation of glia cells [3].

Studies of the genes that play a role in the development of dementia are critically important to understand the mechanism by which this pathology develops. The main genetic factor involved in the development of dementia is the *APOE* ε4 allele, but this allele is neither a necessary nor a sufficient risk factor for this pathology., Recently, other studies have explored other genes such as *CLU*, *PICALM*, and *CR1* [4,5]. Dementia has also been associated with hyperlipidemia [6], while cholesterol is known to play a critical role in the cell membrane [7], whereby excess glucose in serum is broken down via glycation, leading to the formation of advanced glycation end products (AGEs) [8,9].

The receptor for *AGEs* (*RAGE*) is a multiligand signal transduction receptor, with the ability to bind with *AGEs* and β-amyloid (Aβ) [8]. *AGEs* and these ligands have been implicated in the pro-inflammatory and neurotoxic reactions in the brains of dementia patients [10].

Recent studies have pointed out the participation of the *AGEs* gene polymorphism in animal models, showing that the expression of *RAGE* is increased and that if its expression is inhibited, the risk of developing dementia is reduced. On the other hand, these gene variants may affect estrogen receptor signaling and alter the response to estrogen hormones, which could impact brain function and contribute to the development of Alzheimer’s disease [11].

Some evidence suggests that dementia could be related to estrogens (ERD); it has been reported that estrogen concentration is decreased in the brains of women who have dementia [12]. It has also been pointed out that estrogen receptors in the brain influence the development and differentiation of neuronal populations by modulating neurotransmission, neurite sprouting, and axonal growth [13]. Given the key role of estrogen in cholinergic transmission relevant to congestion and mood—association with the development of dementia—and understanding that estrogen activity in the brain is mediated through activation of intracellular, transmembrane, and membrane-bound estrogen receptors (ERs), along with non-genomic mechanisms, certain estrogen receptor α (ESR1) polymorphisms have been linked to an increased risk of cognitive impairment in older women [14,15] and diminution of the cerebral cortex [16]. However, even the exact role of ESR1 in Alzheimer’s disease is still not fully understood and more research is needed to elucidate its precise mechanisms.

Another important component that has been associated with the development of dementia is oxidative stress, which is regulated by *Glutathione S-transferases* (GST) through the maintenance of glutathione levels, allowing for cellular detoxification and activating cellular apoptosis signals [17], in addition to activating proinflammatory cytokines [18]. Therefore, cytokines have been studied in different populations to establish the relationship between the association of Alzheimer’s disease with some SNPs in the *GSTO* locus and late-onset dementia; however, the results remain controversial.

While the genes *ESR1*, *GSTO1*, and *AGER* have been studied in relation to Alzheimer’s disease, the exact mechanisms by which they contribute to the development and progression of the disease are still not fully understood. Further research is needed to elucidate the precise roles of these genes in Alzheimer’s disease and their potential as therapeutic targets.

## 2. Materials and Methods

### 2.1. Study Population

The participants included in this study were selected from the Study of Aging and Dementia in Mexico (SADEM) [19] cohort study carried out in Mexico City from 2010 to date with adults aged 60 years and over. For the diagnosis of dementia the Diagnostic and Statistical Manual of Mental Disorders criteria for dementia (DMS-V) [20] and the National Institute for Neurological and Communicative Disorders and Stroke and the Alzheimer’s Disease and Related Disorders Association (NINCDS-ADRDA) were considered [21] and confirmed by a panel of experts made up of geriatric physicians, neuropsychologists, and neurologists.

The exclusion criteria were previous traumatic brain injury, antipsychotic medication, stroke, and residence in another state. After receiving signed informed consent to participate in the study from the participants or their legal representatives, a face-to-face interview was conducted by trained health personnel to obtain the following information: name, age, sex, birthplace, previous disease history, body mass index (BMI), which was calculated using height and weight, and current alcohol and smoking consumption.

The population included in the present study was made up of 221 adults aged 60 years and older with a confirmed diagnosis of dementia, and 534 subjects with a negative diagnosis for cognitive impairment or any type of dementia who were randomly selected from the participants the SADEM. Study.

### 2.2. Biochemical Analysis of Blood

A venous blood sample was taken from each participant under fasting conditions. These samples were processed in the clinical chemistry laboratory of the National Medical Center, IMSS. The semiautomatic chemical analyzer Ekem Control Lab was used to determine the concentration of high-density lipoprotein cholesterol (HDL-c) and triglyceride in the blood. We used the modified Friedewald formula to determine the concentration of low-density lipoprotein cholesterol (LDL-c) [22].

### 2.3. Genotyping and SNP Selection

The blood samples were used to extract the genetic material (DNA) using the method developed by Lahiri and Numberger [23]. The purity and concentration of the extracted DNA were verified with the NanoDrop^®^ 1000 spectrophotometer (Thermo Fisher Scientific, Wilmington, NC, USA), at a wavelength of 260 and 280 nm, and the extracted DNA was stored at −70 °C until analysis. To select the SNPs for this study, we considered those previously reported in the database website (www.ncbi.nlm.nih.gov/SNP, accessed on 25 May 2023), choosing SNPs that had an LD threshold of r^2^ ≤ 0.1, a 1 Mb interval Hardy–Weinberg equilibrium (HWE) > 0.05, and a minor allele frequency (MAF) ≥ 1%. Finally, a total of three SNPs (rs3844508 in ESR1, rs2070600 in AGEr and rs4925 in GSTO1) were considered for this association study. HaploView 4.2 software was used to analyze genotype data (www.broad.mit.edu/mpg/haploview, accessed on 25 May 2023). As shown in Table 1, we selected the SNPs rs3844508 for ESR1, rs2070600 for AGER, and rs4925 for GSTO1 in the present study.

### 2.4. Multifactor Dimensionality Reduction Analysis

To study epistasis, multifactor dimensionality reduction (MDR) analysis was evaluated in the MDR v3.0.2 statistical package using the Ritchie algorithm. In our study, the MDR was carried out in two steps. First, the best multifactor combination is selected and then the genotype combinations are classified into high and low risk groups to generate models [24]. The best prediction model was selected for maximum training, test balance accuracy, and cross-validation consistency. The model with the values of precision, sensitivity, specificity, and risk was tested using the χ^2^ test at 0.05 significance. Interaction entropy plots were constructed to determine [25].

Entropy graphs comprise nodes that contain the percentage of entropy for each variable, and the values within the nodes indicate the main effects. Positive entropy or interaction are represented by the color plotted in red or orange, while negative entropy plotted in green indicates redundancy. For the MDR model, age was coded as a dichotomous variable with 0 as <70 years and 1 as ≥70 years, the glucose variable 0 as <100 mg/dL and 1 as >100 mg/dL, triglycerides 0 < 150 mg/dL and 1 > 150 mg/dL and the Mini-Mental State Examination MMSE as 0 > 24 points and 1 < 24 points. In the first analysis, the best model was sought by examining the interaction of each polymorphic site and all genes, adjusted for age and cognitive function. In a second analysis, the altered clinical variables were assessed.

### 2.5. Bioinformatics Analysis

Our analysis was performed to examine the association between the loci/genes–proteins involved in dementia. A gene network shwoing the genes of interest was obtained using GeneMANIA [26] (*GSTO1*, *ESR1* and *AGER*), which allowed us to identify genetic interactions, co-expression, co-localization, shared protein domains, and predicted functional relationships between genes and proteins. The size of the nodes is proportional to the weight of the interaction, with scores ranging from 0 and 1 [0 represents a null interaction and 1 represents a strong interaction]. Already known protein–protein interaction (PPI) networks were also analyzed in addition to the computational prediction using the STRING database [27] in 2023.

### 2.6. Statistical Analysis

For the statistical analysis, we used the statistical packages SPSS 25.0 (SPSS, Chicago IL, USA), HaploView program, (www.broad.mit.edu/mpg/haploview, accessed on 12 July 2024) and multifactor dimensionality reduction (MDR version 3.0.2) software (Computational Genetics Laboratory Institute for Quantitative Biomedical Sciences Dartmouth New Hampshire, Lebanon, NH, USA. https://www.epistasis.org, accessed on 12 July 2024) [28]. A *p*-value < 0.05 was considered statistically significant. A baseline analysis was carried out to describe the case and control study groups using the χ^2^ test and Student’s *t*-test. The Hardy–Weinberg equilibrium was determined using the χ^2^ test. We used logistic regression analysis to assess the correlation between genetic variations and the risk factors for dementia, considering odds ratios (OR) and 95% confidence intervals (CI) as indicators.

## 3. Results

### 3.1. Subject Characteristics

This study included 221 cases and 551 controls. Table 2 shows the demographic and clinical characteristics of the study groups. In both groups, women made up the highest proportion (65.6% in cases and 61.9% in controls) and the mean age was higher among cases (76.9 ± 7.9) compared to controls (71.5 ± 7.8). The participants with dementia had a lower educational level (*p* ≤ 0.0001) as well as a higher concentration of glucose, cholesterol, and triglycerides in the blood, with this difference being statistically significant (*p* ≤ 0.0001). On the other hand, diabetes and hypertension were more prevalent among the group of participants with dementia compared to the control group (2.34 (1.70–3.22) *p* = 0.000 1.77 (1.24–2.54) *p* = 0.001 respectively), although more comorbidities and a higher BMI were observed among the case group; these values were not statistically significant. Regarding habits, smoking represented a risk factor for dementia (1.27 (1.17–1.38) *p* = 0.061), though this was not the case for alcohol consumption.

### 3.2. Dementia and the Polymorphisms

The genotype frequencies of the polymorphic studied for *GSTO1*, *ESR1,* and *AGER* are shown in Table 3. The presence of the C allele of the rs4925 and rs2070600 genotypes was significantly higher in cases in comparison with the controls (*p* < 0.05), while the A allele of the rs3844508 genotype occurred more frequently among patients with dementia. We observed associations between the polymorphism of *GSTO1* and risk of dementia for the site rs4925 with the recessive model (OR = 1.720, 95% CI = 1.166–2.537 *p* = 0.006). Similarly, the site *AGER* rs2070600 showed risk of dementia with an additive model of inheritance (OR = 7.278, 95% CI = 3.140–16.868; *p* < 0.001). On the other hand, with respect to ESR1 rs3844508 genotypes, the distribution of the AA genotype was not significantly different in both groups (46.0% in controls versus 55.2% in dementia, *p* = 0.061). The subjects identified as A allele carriers had a lower risk of developing dementia compared to non-carriers, using an over-dominant model (OR = 0.6, 95% CI = 0.45–0.89).

### 3.3. Gene–Gene Interactions to MDR Analysis

The MDR identified the interaction between GSTO1_rs4925, *AGER*_rs2070600, *ESR1*_rs3844508, age, MMSE, triglyceride, HTA, and T2M (*p* < 0.001; OR = 26.662 95% CI (16.164–43.979) as the best prediction model, with an accuracy of 79.6%, a significance of *p* < 0.0001, and a cross-validation of 10/10. The models with four interactions showed a decrease in precision with statistical significance. Only one model showed an increase in sensitivity and specificity. Table 4 shows the result for each SNP. The highest percentage score in the circular diagram was presented for the *AGER*_rs2070600 gene (3.5%) from among all the genetic variants studied.

**Figure 1:**
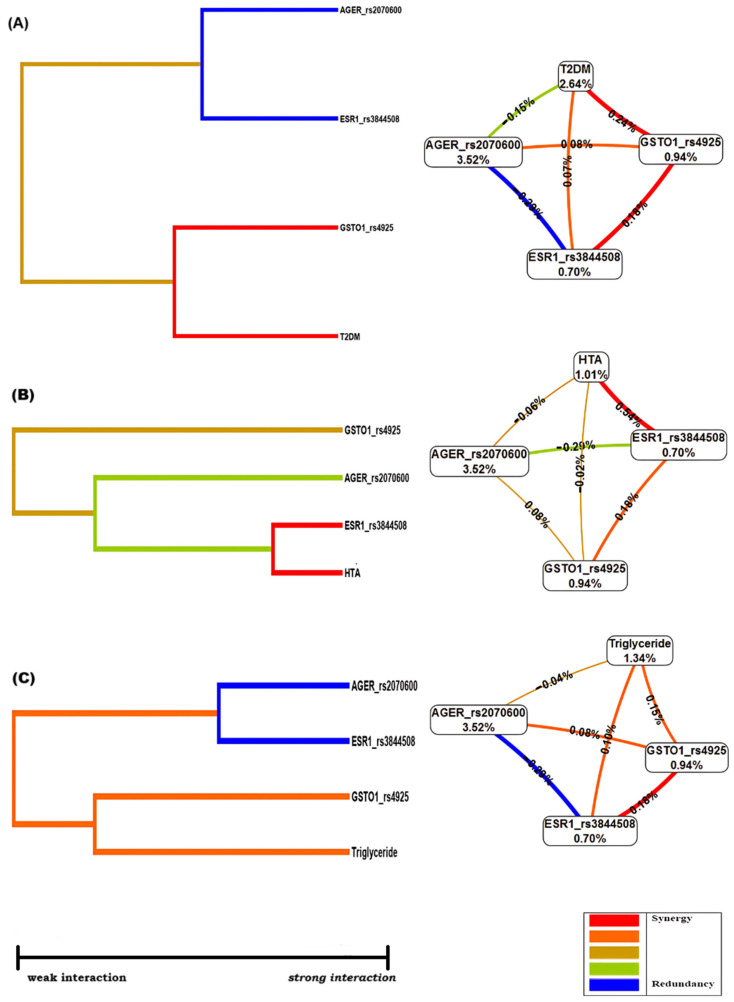
Diagram the MDR Anal-ysis of the ESR1_rs3844508, GSTO1_rs4926, and AGER_rs2070600 poly-morphisms and the risk variables. Dendrogram interaction graphs and circle graph: (**A**) T2M and SNPs, (**B**) HTA and SNPs, and (**C**) Triglycerides and SNPs. The color of the line indicates the type of interaction: red and orange suggest that there is a synergistic relationship (epistasis); yellow suggests independence; and green and blue suggest redundancy or connections.

The most significant contribution is seen marked in red between *ESR1*_rs3844508 and *GSTO1*_rs4925 and is more significant than the individual score for each one (0.18%), as shown in Figure 1A. The factors that increased risk in the interaction of the studied genes were those related to cardiovascular health, such as triglyceride, HBP, and T2M, as observed in the final model.

### 3.4. Prediction of Genetic Functionality

**Figure 2:**
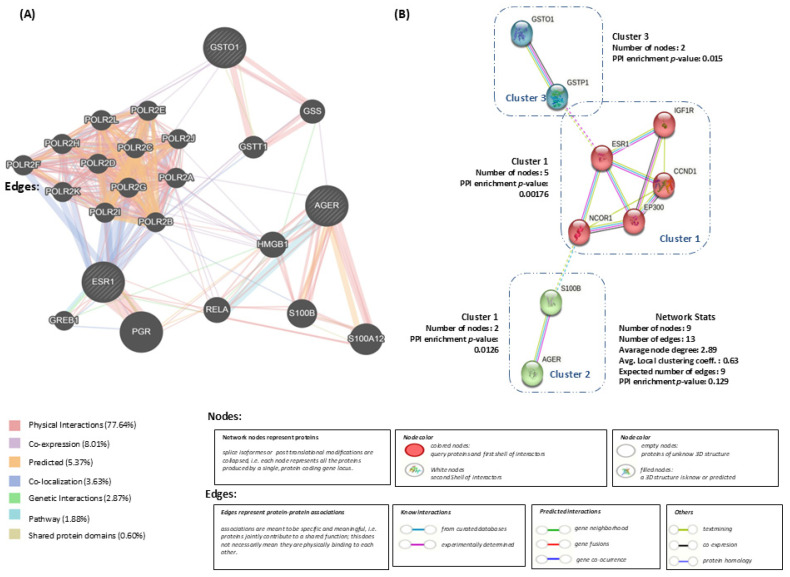
Bioinformatics analysis of *GSTO1*, *ESR1*, and *AGER* genes and proteins for dementia in various tissues/organs. (**A**) Gene–gene interaction networks using GeneMania resource (http://genemania.org (accessed on 19 December 2023); Application version: 3.6.0); (**B**) protein–protein interaction networks using STRING resource (https://string-db.org/) (accessed on 19 December 2023).

Analysis of the *GSTO1*, *ESR1*, and *AGER* genes generated a gene network with a total of 23 nodes and 733 interactions. It shows the physical interactions (77.64%), genetic interactions (2.87%), participation of gene products within the same pathway (1.88%), co-expression (8.01%), co-localization (3.63%), shared protein domains (0.60%), and predicted functional relationships between genes and proteins (5.37%), as shown in Figure 2A. In addition, interactions with other genes of the *AGER* family are shown in *GSTO* and *ESR*. The nodes with stripes inside correspond to the genes of interest. The size of the nodes is proportional to the weight of the interaction, where 1 represents a strong interaction (Supplementary Material S1). For the protein–protein interaction (PPI) with STRING, the number of interacting nodes is 9, with a PPI value *p* = 0.129. The functional enrichment in the network was 146 terms in five categories. Three clusters were formed: Cluster 1 contains five nodes of the proteins CCND1, EP300, ESR1, IGF1R and NCOR1, with an IPP *p* = 0.00176, which are related to biological processes involving response to estrogens and regulation of the androgen receptor signaling pathway. Cluster 2 contains two nodes of the proteins AGER and S100B, which are related to the advanced signaling of the glycosylation end product receptor, with a PPI *p* = 0.0126. Cluster 3 contains two nodes of the proteins GSTO1 and GSTP1, which are related to the biosynthetic process derived from glutathione, with a PPI *p* = 0.015 (Figure 1B) (Supplementary Material S2 Excel).

## 4. Discussion

Currently there is no treatment to stop dementia, so the search for new factors at both the genetic and environmental levels can provide new information that allows for the development of effective diagnostic strategies and future treatments. In this study, we have used bioinformatic analysis and the MDR analysis to identify patterns of interaction between the genetic associations [29].

The findings of this study confirmed intergenic interactions of the SNPs *GSTO1*_rs4925, *AGER*_rs2070600, and *ESR1*_rs3844508 with variables such as age, MMSE evaluation of cognitive status, high triglycerides, and having HTA and T2M, showing a higher risk for susceptibility to dementia OR = 26.662 95% CI (16.164–43.979) with *p* < 0.001. These findings are relevant since previously reported results for these polymorphisms were controversial [30,31,32]; these inconsistencies can be attributed to the small sample sizes and the methodologies used.

Our results showed that *GSTO1*_rs4925 was associated with the risk of developing dementia when the genotype AA was present, while the presence of the C allele seemed to show protection against the development of dementia. These results have been reported in very few works carried out in other populations [32,33]. Therefore, our results contribute to the understanding of the role that *GSTO1* plays in cellular detoxification, with its main function being the elimination of toxic compounds from the body. As an oxidant, it helps neutralize free radicals, thereby protecting neurons by acting as a significant regulator and cellular signaling modulator.

On the other hand, for *AGER*-rs2070600, we identified a risk association for dementia with the CC alleles (OR = 7.27 95% CI 3.14–16.8) *p* < 0.001) [11,34]. These results have been controversial in previously reported studies. Therefore, the association that we identified may help us understand the role that the RAGE gene plays in the development of dementia, whether its product participates in inflammatory processes, and the mechanisms involved in oxidative stress. The variation in the production of this enzyme could be modulate the transport of Aβ across the blood–brain barrier [11,35,36]. However, we cannot compare the results in relation to the genetic interaction of *AGER* and other polymorphisms because they have not been reported; however, we can highlight that it could be used as a marker of the disease as it can be identified in fluids such as serum [37].

Finally, the studies carried out on the *ESR1* rs3844508 polymorphism have been conclusive. In our study, we observed that the G allele of the *ESR1* rs3844508 marker was associated with a lower risk for dementia [38]. This association remained significant after the interaction with the *AGER* and *GSTO1* genes.

The expression of this gene, when modified by the variation in the gene, could modulate neuronal functions affecting both neurotransmission, axonal growth, and neuroplasticity [39], which could explain why this polymorphism might be linked to the development of cognitive impairment.

As mentioned previously, this is the first study that relates the genes that participate in the processes related to oxidative stress and the inflammatory response (*GSTO*, *AGER* and *ESR1*) in dementia. Our data show, in addition to an independent risk association between genes, a synergic association that may suggest their necessary involvement in the development of dementia in our population.

It should be noted that both MDR analysis and computer tools today allow us to investigate the simultaneous effects between loci and their products and thus identify risk patterns in multifactorial pathologies such as dementia.

Therefore, we can point out that subjects carrying the combination identified within the highest genetic risk model identified in this study will be more susceptible to developing dementia in relation to those who do not carry it. This could be related to the pathways in which the products of these genes interact, which are related to the management of oxidative stress and cellular detoxification in the brain, which limit the accumulation of Aβ deposits and consequently induce neuronal death.

The ethnic characteristics of our population make these results relevant and allow us to lay the foundation for the identification of new diagnostic markers as well as possible therapeutic targets.

## 5. Conclusions

Our results show that the *GSTO1*_rs4925 and *AGER*_rs2070600 gene loci are associated with CC susceptibility to dementia. This study also highlights the protective role of *ESR1*_rs3844508 polymorphism in dementia predisposition. These findings provide new biological information for assessing dementia risk, allowing us to complement its pathogenesis and generating information for the development of new diagnostic strategies and treatments.

## Figures and Tables

**Table 1:** Description genetic variants studied.

Gene	Name	dbSNPb	Allele Change (Ancestral Allele)	Chr. Position (bp)	Consequence
*ESR1*	Estrogen receptor α	rs3844508	A > G	chr6:151818907	Intron Variant
*AGER*	Advanced glycosylation end-product specific receptor	rs2070600	C > T	chr6:32183666	Missense Variant
*GSTO1*	Glutathione S-transferase omega 1	rs4925	C > A	10:104263031	Missense Variant

bSNP ID in database dbSNP. Given name according to NCBI.

**Table 2:** Characteristics of the study groups and risk values.

Variables		All (n = 755)	Dementia (n = 221)	Controls (n = 534)	OR (95% CI)	*p* Value
Gender, n (male%/female%)		279 (36.9)/476(63.1)	76 (34.39)/145(65.61)	203 (38.01)/331(61.99)	0.85 (0.61–1.18)	0.348
Age, years (SD)		73 ± 8.25	76.9 ± 7.9	71.5 ± 7.8	1.08 (1.06–1.10)	<0.001
Education, years (SD)		6.6 ± 5.4	5.7 ± 5.5	6.9 ± 5.2	1.04 (1.01–1.07)	0.006
Glucose (mg/dL)		114.8 ± 44.2	127 ±48.5	109.7 ± 41.3	1.00 (1.00–1.01)	<0.001
Total cholesterol (mg/dL)		215.6 ± 43.6	247 ± 31.6	202.6 ± 41.3	1.03 (1.02–1.03)	<0.001
HDL-cholesterol (mg/dL)		51.4 ± 13.1	48 ± 9.8	52.8 ± 14	0.97 (0.95–0.98)	<0.001
LDL-cholesterol (mg/dL)		121.6 ± 37.7	127.6 ± 40.2	119.1 ± 36.3	1.00 (1.00–1.00)	0.006
Triglycerides (mg/dL)		172.6 ± 87.9	190.8 ± 85.1	165 ± 88	1.00 (1.00–1.00)	<0.001
Charlson comorbility index, n (%)						
	0–1	154 (20.4)	36 (16.3)	118 (22.1)	1.0	
	2–3	321 (42.5)	87 (39.4)	234 (43.8)	1.21 (0.77–1.90)	0.386
	>4	280 (37.9)	98 (44.3)	182 (34.1)	1.76 (1.12–2.75)	0.013
MMSE, (mean ± SD)		24.28 ± 4.21	21.47 ± 3.84	25.45 ± 3.78	1.31 (1.24–1.38)	<0.001
T2M, n (%)		306 (40.53)	122 (55.2)	184 (34.46)	2.34 (1.70–3.22)	0.000
HTA, n (%)		510 (67.55)	168 (76.02)	342 (64.04)	1.77 (1.24–2.54)	<0.001
BMI (kg/m^2^)		27.2 ± 5.42	26.92 ± 6.17	27.3 ± 5.09	0.98 (0.95–1.01)	0.334
Smoker status, n (%)		478 (63.3)	152 (68.8)	326 (61.1)	1.27 (1.17–1.38)	0.061
Alcohol consumption, n (%)		221 (29.3)	43 (19.5)	178 (33.4)	0.48 (0.33–0.70)	<0.001

MMSE, Mini-mental state examination; HTA, hypertension; T2M, type 2 diabetes; OR, odds ratio; CI, confidence interval; *p*-value < 0.05.

**Table 3:** Allele and genotype distribution and model inheritance of polymorphisms in dementia.

Polymorphism	Allele Frequency; n (%)		Genotype Frequency; n (%)			Model Inheritance	** OR (95%CI)	*p*-Value
*GSTO1*-rs4925	C	A	CC	AA	CA			
Controls	0.12	0.87	28 (0.05)	369 (0.69)	137 (0.25)	Codominant	Ref	
Dementia	0.18	0.81	13 (0.05)	177 (0.80)	31 (0.14)		0.99 (0.49–2.01)	0.987
							0.49 (0.22–1.08)	0.077
						Dominant	0.86 (0.42–1.73)	0.676
						Recessive	1.72 (1.16–2.53)	0.006
						Over-dominant	0.49 (0.31–0.76)	0.002
						Additive	0.49 (0.30–0.72)	0.002
*AGER*-rs2070600	C	T	CC	TT	CT	Additive		
Controls	0.99	0.00	52 (0.98)	1 (0.00)	6 (0.01)	Codominant	Ref	
Dementia	0.94	0.05	19 (0.88)	0	25 (0.11)		10.76 (4.23–27.35)	<0.001
							---	--
						Dominant	9.02 (3.74521.76)	<0.001
						Recessive	--	--
						Over-dominant	10.78 (4.24–27.41)	<0.001
						Additive	7.27 (3.14–16.86)	<0.001
*ESR1*-rs3844508	A	G	AA	GG	AG		OR (95% CI)a	*p* Value
Controls	0.72	0.27	247 (0.46)	44 (0.082)	243 (0.45)	Codominant	Ref	
Dementia	0.69	0.31	122 (0.55)	22 (0.09)	77 (0.34)		1.09 (0.61–1.96)	0.750
							0.64 (0.45–0.91)	0.014
						Dominant	0.71 (0.51–0.98)	0.043
						Recessive	1.32 (0.76–2.32)	0.318
						Over-dominant	0.64 (0.45–0.89)	0.009
						Additive	0.80 (0.68–0.96)	0.010

** ORs of the inheritance models are adjusted for age; 95% CI, confidence interval; *p*-value < 0.05.

**Table 4:** The best model for predicting the interaction between dementia and associated genetic variants and environment.

Model	Accuracy	Sensitivity	Specificity	OR (95%CI)	*p*-Value
Interaction Gen-Gen and impaired cognitive function (MMSE)					
*ESR1*_rs3844508, Age, MMSE	0.72	0.73	0.72	7.39 (5.09–10.75)	<0.001
*GSTO1*_rs4925, Age, MMSE	0.69	0.77	0.65	6.79 (4.62–9.97)	<0.001
*AGER*_rs2070600, Age, MMSE	0.69	0.81	0.64	7.65 (5.12–11.42)	<0.001
*GSTO1*_rs4925, *AGER*_rs2070600, *ESR1*_rs3844508, Age, MMSE	0.73	0.75	0.72	8.08 (5.52–11.82)	<0.001
Interaction between genes, impaired cognitive function, and altered clinical variables					
*GSTO1*_rs4925, *AGER*_rs2070600, *ESR1*_rs3844508, Age, MMSE, Glucose, Triglyceride	0.75	0.83	0.71	13.25 (8.64, 20.32)	<0.001
Interaction between genes, impaired cognitive function, altered clinical variables, and comorbidities					
*GSTO1*_rs4925, *AGER*_rs2070600, *ESR1*_rs3844508, Age, MMSE, Glucose, Triglyceride, HTA	0.79	0.86	0.76	19.81 (12.5–31.19)	<0.001
*GSTO1*_rs4925, *AGER*_rs2070600, *ESR1*_rs3844508, Age, MMSE, Triglyceride, HTA, T2M	0.79	0.89	0.75	26.66 (16.16–43.97)	<0.001

ESR1, Estrogen receptor α 1; GSTO1, Glutathione S-transferase omega 1; AGER, advanced glycosylation end-product specific receptor; MMSE, mini-mental state examination; HTA, hypertension; T2M, Type 2 diabetes; OR, odds ratio; CI, confidence interval; *p*-value < 0.05.

## Data Availability

The data presented in this study are available on request from the corresponding author due to ethical restrictions.

## Supplementary Materials

The following supporting information can be downloaded at: https://www.mdpi.com/article/10.3390/genes15111395/s1, Supplementary S1: GeneMANIA report; Supplementary S2: interaction netwoks inferred using STRING source.
